# *Eurosurveillance* reviewers in 2018

**DOI:** 10.2807/1560-7917.ES.2019.24.2.1901102

**Published:** 2019-01-10

**Authors:** Eurosurveillance editorial team

**Affiliations:** 1 European Centre for Disease Prevention and Control (ECDC), Stockholm, Sweden

The editorial team is grateful to all the experts who dedicated their time to peer-review our articles over the past 12 months. We wish to thank them and all those whose names are not listed below but gave helpful advice and support. We apologise to any reviewers whose names we may have missed.

Our reviewers in 2018 were:

Preben Aavitsland; Winston Abara; Mark Achtman; Blythe Adamson; Karam Adel Ali; Amos Adler; Cornelia Adlhoch; Tobias Alfven; Franz Allerberger; Matthias an der Heiden; Nick Andrews; Augustina Annan; Alberto Antonelli; Julien Arino; Laurence Armand; Olle Aspevall; Agoritsa Baka; Biswajit Banik; Lucie Bardet; Ian Barr; Joel Barratt; Mette Bartels; Luisa Barzon; Elizabeth Beech; Michael Behnke; Romeo Bellini; Edward Belongia; Beatrice Bercot; Anne Berger-Carbonne; Dag Berild; Kathy Bernard; Xavier Bertrand; Marianne Besnard; John Besser; Janneke Bil; Zeno Bisoffi; Kathe E Bjork; Dominique Blanc; Aurélie Bocquier; Pierre-Yves Boelle; Shelly Bolotin; Magnus Boman; Paolo Bonanni; Robert A. Bonomo; Marc Bonten; Michael Borg; Maria Borrego; Arnold Bosman; Elisabeth Botelho-Nevers; Julie Bottero; Charles Boucher; Nathalie Boulanger; Herve Bourhy; Naomi Boxall; Jean-Philippe Brandel; Elizabeth Brickley; Tom Britton; Eeva Broberg; Stef Bronzwaer; Tal Brosh-Nissimov; Florence Brossier; Kevin Brown; Mia Brytting; Udo Buchholz; Ann Burchell; Zahid Butt; Valentina Cambiano; Rosa Cano; Gioia Capelli; Saulius Caplinskas; Alessandra Carattoli; Yehuda Carmeli; João André Carriço; Pamela Cassiday; Nadim Cassir; Alejandra Castanon; Vicki Chalker; Meera Chand; Herve Chaudet; Shahnaz Akhtar Chaudhry; Sheng Chen; Gerardo Chowell; Dimple Chudasama; Andrew Clark; Jan Clement; Michelle Cole; Simon Collin; Victor Corman; Helena Cortes Martins; Suzanne Cotter; Simon Cottrell; Suzie Coughlan; Natacha Couto; Susan Cowan; Benjamin Cowling; Anne Claude Cremieux; Natasha Crowcroft; Fortunato D'Ancona; Viktor Dahl; Tim Dallman; Eric Dannaoui; Birgitta de Jong; Gaston De Serres; Henry de Vries; Katja de With; Aleksander Deptula; Tarik Derrough; Jean-Claude Desenclos; Ram Dessau; Joanne Dillon; Roger Dodd; Dragoslav Domanovic; Lisa Domegan; Christina Domingo; Michel Drancourt; Mike Drebot; Erika Duffell; Tim Eckmanns; Paul Effler; Carina Eklund; Sascha Ellington; Delia Enria; Onder Ergonul; Nagy Erzsébet; Concepcion Estivariz; Martin Exner; David Eyre; Anna-Bella Failloux; Christopher Fairley; Gerhard Falkenhorst; Heinz Feldmann; Peter Feng; Olivier Ferraris; Marc Fischer; Julia Fitzner; Brendan Flannery; Stefan Flasche; Agnes Fleury; Ana Gloria Fonseca; Ivo Foppa; Pieter Fraaij; Christina Frank; Eelco Franz; Scott Fridkin; Hilbert Friederike; Alexander Friedrich; Ingrid Friesema; Niels Frimodt-Möller; Angelika Fruth; Giovanni Gabutti; Pierre Gallian; Giuliano Garofolo; Philippe Gautret; Julianne Gee; Peter Gerner-Smidt; Michael Geschwind; Tommaso Giani; Christopher Gilpin; Christophe Ginevra; Aharona Glatman-Freedman; Anja Globig; Y Glupczynski; Pere Godoy; Daniel Golparian; Douglas Goodin; Ewelina Gowin; Luigi Gradoni; Christina Greenaway; Lutz Guertler; Barbara Gunsenheimer-Bartmeyer; Bernardo Rafael Guzman Herrador; Huldrych Günthard; Bart Haagmans; Katrine Bach Habersaat; Marisa Haenni; Susan Hahne; Ágnes Hajdu; Sebastian Haller; Anette Hammerum; Håkan Hanberger; Stephan Harbarth; Pia Hardelid; Sally Hargreaves; Heli Harvala; Jaythoon Hassan; Barbara Hauer; Joana Haussig; Peter Helbling; Rene Hendriksen; Niel Hens; Bjorn Herrmann; Sereina Herzog; Kristen L Hess; Ursel Heudorf; Ole Heuer; Roger Hewson; Lauri Hicks; Andy IM Hoepelmann; Sven Hoffner; Michael Hogardt; Kristy Horan; Te-Din Huang; Yvan Hutin; Michael Höhle; Giovanni Ianiro; Derval Igoe; Giuseppe Ippolito; Jennifer Isenor; Michael Jackson; Lynn Janssen; Claire Jenkins; Seok Hoon Jeong; Samo Jeverica; Silvia Jimenez-Jorge; Kari Johansen; Alan Johnson; Helen Johnson; Pikka Jokelainen; Michael Jordan; Loic Josseran; Achim Kaasch; Sarah Kabbani; Annemarie Kaesbohrer; Bernard Kaic; Ilias Karaiskos; Tommi Karki; Peter Keller; Volkhard Kempf; Yury Khudyakov; Mirjam Knol; Anke Kohlenberg; Axel Kola; Gerard Krause; Peter Krause; Peter Kreidl; Sanjeev Krishna; Paul Krogstad; Anna Kuehne; Katrin Kuhn; Ed Kuijper; Heinke Kunst; Robin Köck; Angie Lackenby; Shamez Ladhani; John Lambert; Amparo Larrauri; Heidi Larson; Simon Le Hello; Carol Leitch; Tinne Lernout; Joseph Lewnard; Bruno Lina; Andrew Lloyd; Shawn Lockhart; Nicholas Loman; Pierluigi Lopalco; Jens Lundgren; Yaniv Lustig; Michael S. Lyons; Outi Lyytikäinen; Daniel Lévy-Bruhl; Noni Mac Donald; Kristine Macartney; Liane Macdonald; Colin Mackenzie; Shelley S. Magill; Anna-Pelagia Magiorakos; Barbara Mahon; Helena Maltezou; Jean-Michel Mansuy; Ulrich Marcus; Lauri E. Markowitz; Nicolee Martin; Clara Marín; Maria Teresa Mascellino; Virginie Masserey; Sarah Mbaeyi; Scott McDonald; Jeffrey McFarland; Peter McIntyre; Huong McLean; Jim McMenamin; Nicholas Medland; Conor Meehan; Adam Meijer; Elissa Meites; Angeliki Melidou; Kassiani Mellou; Ella Mendelson; Jeffrey W. Mercante; Mathias Merker; Stefano Merler; Jaimie Meyer; Claire Midgley; Sofie Midgley; Andrei Mihalca; Massimo Mirandola; Hamish Mohammed; Orna Mor; Dearbhaile Morris; Anne Mosnier; Joël Mossong; Judith Mueller; Sanjin Musa; Didier Musso; Luise Müller; Rajeshwari Nair; Dilip Nathwani; Warrick Nelson; Lina Nerlander; Emanuele Nicastri; Matthias Niedrig; Harold Noel; Paulo Nogueira; Torbjörn Norén; Hans-Dieter Nothdurft; Norbert Nowotny; Baltazar Nunes; Claudia Nunes Duarte dos Santos; Mari Nygård; Colm A. O'Morain; Sophie Octavia; Nicholas Ogden; Isabel Oliver; Jesús Oteo; W John Paget; Ana M. Palomar; Constantinos Papagiannitsis; Dimitrios Paraskevis; Cyra Patel; John Paul; Lucia Pawloski; Lara Payne; Malik Peiris; Camille Pelat; Pasi Penttinen; Freddy Perez; Jeannine Petersen; Lyle Petersen; Martin Petric; Juraj Petrik; Yvonne Pfeifer; Anastasia Pharris; Nick Phin; Mathieu Picardeau; Roan Pijnacker; Tamara Pilishvili; Alessandro Pini; Jillian Pintye; Diamantis Plachouras; Laurent Poirel; Jeanine Pommier; Garyfallia Poulakou; Jan Prins; Katarina Prosenc; Andrea Pugliese; Celine Pulcini; David W Purcell; Helen Quinn; Sophie Quoilin; Veronique Raimond; Mario Ramirez; Brian Raphael; Vincenza Regine; Renata Reis; Chantal Reusken; Sandra Reuter; Giovanni Rezza; Anders Rhod Larsen; Dale Rhoda; Caterina Rizzo; Emmanuel Robesyn; Eve Robinson; Jesús Rodríguez-Baño; T Rolling; Laurence Roope; Roberto Rosa; John Rossen; Mirko Rossi; Gian Maria Rossolini; Adam Roth; Maja Rupnik; Werner Ruppitsch; Claudia Ruscher; Kristi Rüütel; Joaquin Salas-Coronas; David Samara; Jussi Sane; Sabine Santibanez; Loredana Sarmati; Andre Sasse; Silvia Sauleda Oliveras; Gianpaolo Scalia-Tomba; Gaia Scavia; Robert Schlaberg; Patricia Schlagenhauf; Jonas Schmidt-Chanasit; Eli Schwartz; Kevin Schwartz; Torsten Semmler; William Shafer; Ian Simms; Benedetto Simone; Martin Singer; Vitali Sintchenko; Teemu Smura; Heidi Soeters; Hanna Soini; Carla A. Sousa; Christina Spiropoulou; Gianfranco Spiteri; Russell Stafford; Helen Stagg; Gerold Stanek; Pawel Stefanoff; Claudia Stein; Chen Stein-Zamir; Rune Stensvold; Maja Subelj; Carl Suetens; Kathryn Suh; Jonathan Suk; Sheena Sullivan; Arnfinn Sundsfjord; Johanna Takkinen; Arnaud Tarantola; Anders Tegnell; Noemia Teixeira de Siqueira-Filha; Laura Temime; Nicola Thompson; Claire Thorne; Salla Toikkanen; M Tongo; Mia Torpdahl; Soren Uldum; Tim Uyeki; Palle Valentiner-Branth; Snigdha Vallabhaneni; Alex van Belkum; Wim Van Bortel; Marita van de Laar; Kees. C. van den Wijngaard; Arie van der Ende; Maarten F. Schim Van Der Loeff; Marianne van der Sande; Marieke J. van der Werf; Gary Van Domselaar; Sofie van Dorp; Jakko Van Ingen; Olli Vapalahti; Carmen Varela Martinez; Alkiviadis Vatopoulos; Alida Veloo; Harry Vennema; Salvador Vergara-López; Pierre Verger; Lasse Vestergaard; Jan Vinjé; Leo Visser; Margreet Vos; Elena Vovc; Julio A. Vázquez Moreno; Timothy Walsh; Jan Walter; Doreen Walther; Jiangrong Wang; Francois Xavier Weill; Hillard Weinstock; Dirk Werber; Guido Werner; Henrik Westh; Therese Westrell; David Whiley; Ole Wichmann; Andreas Widmer; Annelies Wilder-Smith; Hendrik Wilking; Christopher Williams; Carl-Heinz Wirsing von König; Edwina Wright; Lin Yang; Abdool Yasseen; Hans L Zaaijer; Katherina Zakikhany; Philipp Zanger; Gernot Zarfel; Herve Zeller; Walter Zingg; Ines Zollner-Schwetz; Huachun Zou; Phillip Zucs.

